# Natural killer cell immunotherapy in glioblastoma

**DOI:** 10.1007/s12672-022-00567-1

**Published:** 2022-10-28

**Authors:** Hamed Hosseinalizadeh, Mehryar Habibi Roudkenar, Amaneh Mohammadi Roushandeh, Yoshikazu Kuwahara, Kazuo Tomita, Tomoaki Sato

**Affiliations:** 1 Department of Medical Biotechnology, Faculty of Paramedicine, Guilan University of Medical Sciences, Rasht, Iran; 2 Burn and Regenerative Medicine Research Center, School of Medicine, Velayat Hospital, Guilan University of Medical Sciences, Rasht, Iran; 3 Department of Applied Pharmacology, Graduate School of Medical and Dental Sciences, Kagoshima University, Kagoshima, Japan; 4 Division of Radiation Biology and Medicine, Faculty of Medicine, Tohoku Medical and Pharmaceutical University, Sendai, Japan

**Keywords:** Glioblastoma, NK cells, Immunotherapy

## Abstract

Glioblastoma (GBM) is one of the most difficult cancers to treat because GBM has the high therapeutic resistance. Recently, immunotherapies for GBM have been used instead of conventional treatments. Among them, Natural killer (NK) cell-based immunotherapy has the potential to treat GBM due to its properties such as the absence of restriction by antigen-antibody reaction and deep penetration into the tumor microenvironment. Especially, genetically engineered NK cells, such as chimeric antigen receptor (CAR)-NK cells, dual antigen-targeting CAR NK cells, and adapter chimeric antigen receptor NK cells are considered to be an important tool for GBM immunotherapy. Therefore, this review describes the recent efforts of NK cell-based immunotherapy in GBM patients. We also describe key receptors expressing on NK cells such as killer cell immunoglobulin-like receptor, CD16, and natural killer group 2, member D (NKG2DL) receptor and discuss the function and importance of these molecules.

## Introduction

Glioblastoma (GBM) is the most common primary brain cancer and accounts for nearly 50% of mortality and morbidity of all brain cancers. Despite the improvement in the diagnosis and therapeutic agents for GBM are being developed one after another, survival rates are insignificant. Only 25% of patients live more than one year, and 5% live more than five years [1]. The current treatments for GBM, which include combinations of surgery, chemotherapy, and radiotherapy, have remained unchanged since 2005, yet statistical results suggest that these techniques have failed to increase the therapeutic efficiency and prognosis of GBM patients [2].

Recently, numerous studies have reported optimistic outcomes for the role of immunotherapy in treating GBM including an increase of 5-year survival by almost 13% compared to 5% for conventional treatment strategies [3]. One aspect of cancer immunotherapy, sometimes referred to biological therapy, is the engineering of immune cells to boost the body’s natural defenses against cancer. However, due to the exclusive properties of glioma cells, immunotherapy has faced multiple challenges such as an immunosuppressive microenvironment, low immunogenicity, strong heterogeneity, and escape from immune surveillance [4, 5]. As a result, researchers have struggled to determine the effectiveness of immunotherapy in GBM. Though still an emerging technique, among other lymphocytes, natural killer (NK) cells may be more appropriate as therapeutic agents against the microenvironment and heterogeneous characteristics of GBM. Due to their properties, such as resistance to immune suppression and deep penetration into the tumor microenvironment, genetically engineered NK cells may become an important tool for GBM immunotherapy [5]. Here, we review recent efforts in the use of NK cell-based immunotherapy in GBM patients.

## Chimeric antigen receptor-NK cells (CAR-NK cells)

**Fig. 1:**
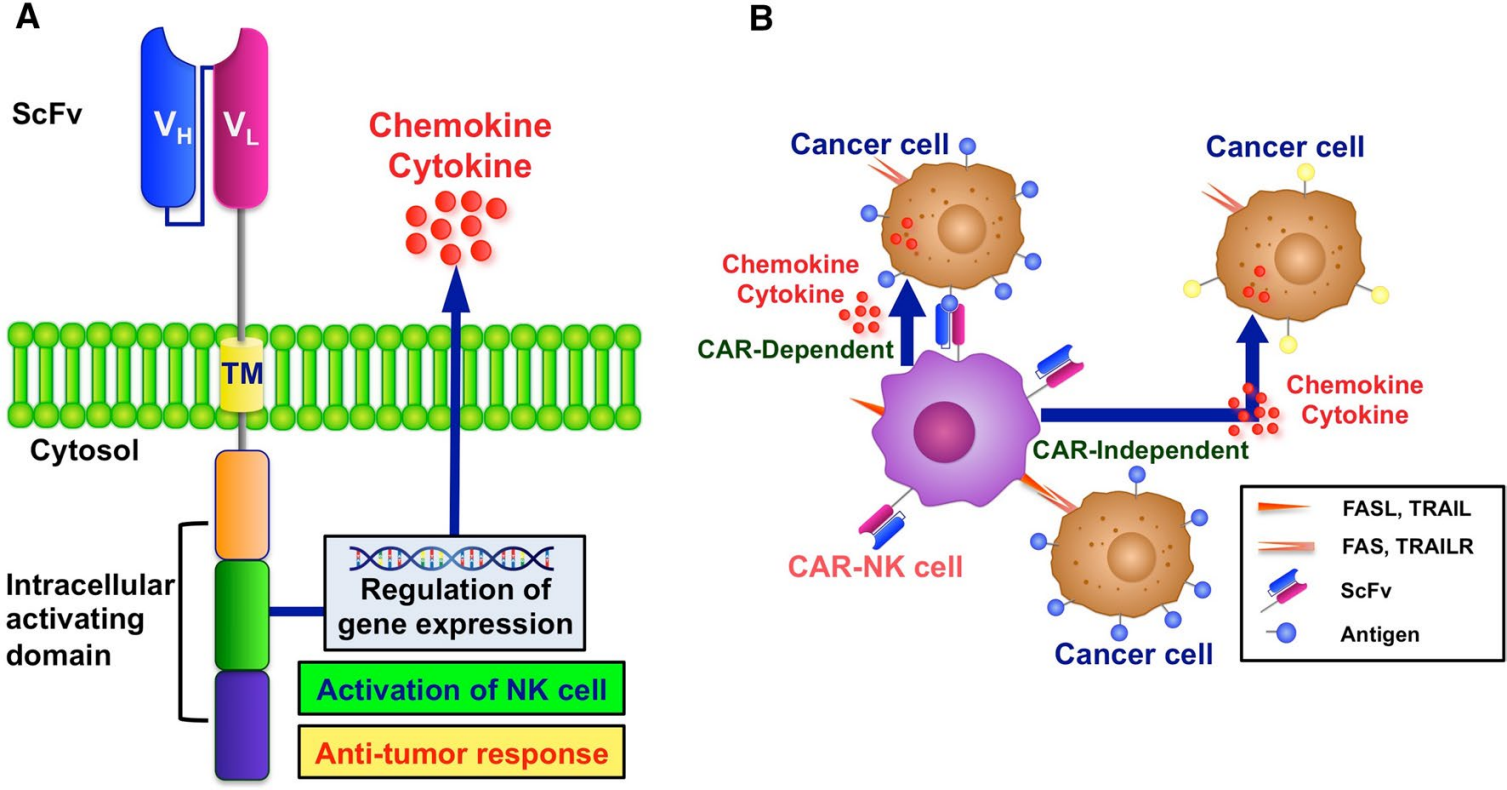
Structure of chimeric antigen receptor (CAR)- Natural Killer (NK) cells and the manner of tumor cell elimination. **A** Functional structure of CAR. CAR consists of three major sites: the extracellular region with a single-chain variable fragment (scFv), the intracellular region with a signal activating domain, and the transmembrane (TM) region that connects the extracellular and intracellular regions. ScFv recognizes tumor antigens that are overexpressed in cancer cells or expressed only in cancer cells. The TM region transduces activation signals recognized by scFv into intracellular activation signals. The intracellular activating domain regulates gene expression, which activates NK cells and stimulates chemokine and cytokine secretion. **B** The CAR-dependent or CAR-independent manner of tumor cell elimination. CAR-NK cells recognize tumor antigens and activated themselves. Activated CAR-NK cells produce secretory cytokines and chemokines. These are effective not only on CAR-recognized cells (CAR-dependent) but also on cancer cells that do not express the tumor antigens recognized by scFv (CAR-independent). Activated CAR-NK cells express FAS ligand (FASL) or TRAIL. FAS and TRAILR recognize FASL and TRAIL on the surface of cancer cells, respectively. These promote cancer cell apoptosis in a CAR-independent manner

Similar to the efficiency and effectiveness of CAR-engineered T cells in the treatment of GBM recurrence, the adoptive transfer of CAR-modified NK cells has shown an appreciable anti-glioma activity both in vitro and *in vivo.* However, in contrast to CAR-T cells, which require an autologous supply for each patient, NK cells are safe in an allogeneic condition, which broadens the range of cell donors for generating clinically relevant doses of CAR-NK cells for therapy [6]. Moreover, CAR-NK cells are superior to CAR-T cells in safety because they act free from the antigen-antibody reaction and do not induce a cytotoxic effect such as cytokine release syndrome using a variety of tests [7]. Therefore, the intrinsic properties of NK cells make them an appealing alternative as CAR-engineered effectors in cancer treatment. This has paved the way for several clinical trials to be conducted to develop this method further and improve its ability to act against glioma cells [8, 9]. Briefly, CAR-NK cells can recognize CAR-targeted antigens and stimulate NK cell activation, proliferation, and secretion of various inflammatory cytokines and chemokines. Following cancer cell recognition, NK cells form a lytic synapse between themselves and cancer cells to enable guided delivery of lytic granules against susceptible cancer cells while still retaining their natural activating and inhibitory receptors [7]. Thereby, CAR-NK cells, in addition to eliminating tumor cells in a CAR-dependent manner, can destroy cancer cells that do not express CAR-targeted antigens (CAR-independent) (Fig. 1) [10].

CARs are made of a modified fusion protein based on the T cell receptor. It incorporates single-chain variable fragment (scFv/FRP5) as an extracellular antigen-binding domain that is predetermined to identify tumor antigens overexpressed on or unique to cancerous cells (in a major histocompatibility-independent way) and is connected to an intracellular activation signaling domain(s). CAR activation by a particular antigen leads to activation of the downstream signaling pathway that assists in the killing of malignant cells [11, 12]. Molecular research has shown that the epidermal growth factor receptor (EGFR) and its mutant version, EGFR variant III (EGFRvIII), are often overexpressed in GBM patients. Müller et al. developed a particular CAR on the cell surface of modified NK cells for the treatment of GBM that improved glioma cells destruction and increased survival rates in mice harboring GBM cancer [13]. The CAR consisted of a portion of the EGFRvIII-specific antibody (also called MR1) as a single-chain variable fragment (scFv) that was linked to the DNAX-activating protein 12 (DAP12) domain as an intercellular region for signal transduction, resulting in increased cytotoxicity to cancer cells, the release of pro-inflammatory factors, and extend symptom-free survival in immunocompetent mice [5, 13, 14]. DAP12 is a protein with three immunoreceptor tyrosine-based activation motifs that transmits signals via activated NK cell receptors [13]. Other studies have demonstrated that additional alterations of NK cells to carry an MR1 antibody with C-X-C motif chemokine receptor 4 increased the chemotaxis to C-X-C motif chemokine 12, also known as stromal cell-derived factor 1 generated by glioma cells, leading to higher stability in the circulation compared to NK cells expressing EGFRvIII alone [13].

Similar to EGFRvIII, human epidermal growth factor receptor 2 (HER2/ErbB2) has been proposed as a potential candidate for a tumor antigen. It belongs to a subfamily of EGFR and is overexpressed in 80% of tumor samples of GBMs and is associated with poor survival [15]. Zhang et al., developed genetically engineered NK cells to express a CAR that consisted of the HER2-specific antibody FRP5 and related intracellular signaling domains Cluster of Differentiation 28 (CD28) and CD3ζ [16]. This study indicated the higher anti-cancer effects of ErbB2-specific CAR NK cells against glioma cells, which minimized the spread of tumors in immunocompetent mice [16, 17]. However, immunological evasion by antigen-loss variations as well as the heterogenic characteristics of GBM clones considered as the primary causes of CAR-NK cell failure would limit their use in clinical application. As a result, there is an urgent need for next-generation therapies to address these newly identified hurdles. Preclinical studies have examined methods such as dual antigen-targeting and adapter chimeric antigen receptors (AdCAR) to overcome this resistance to treatment [18, 19].

## Dual antigen-targeting CAR NK cells

**Fig. 2:**
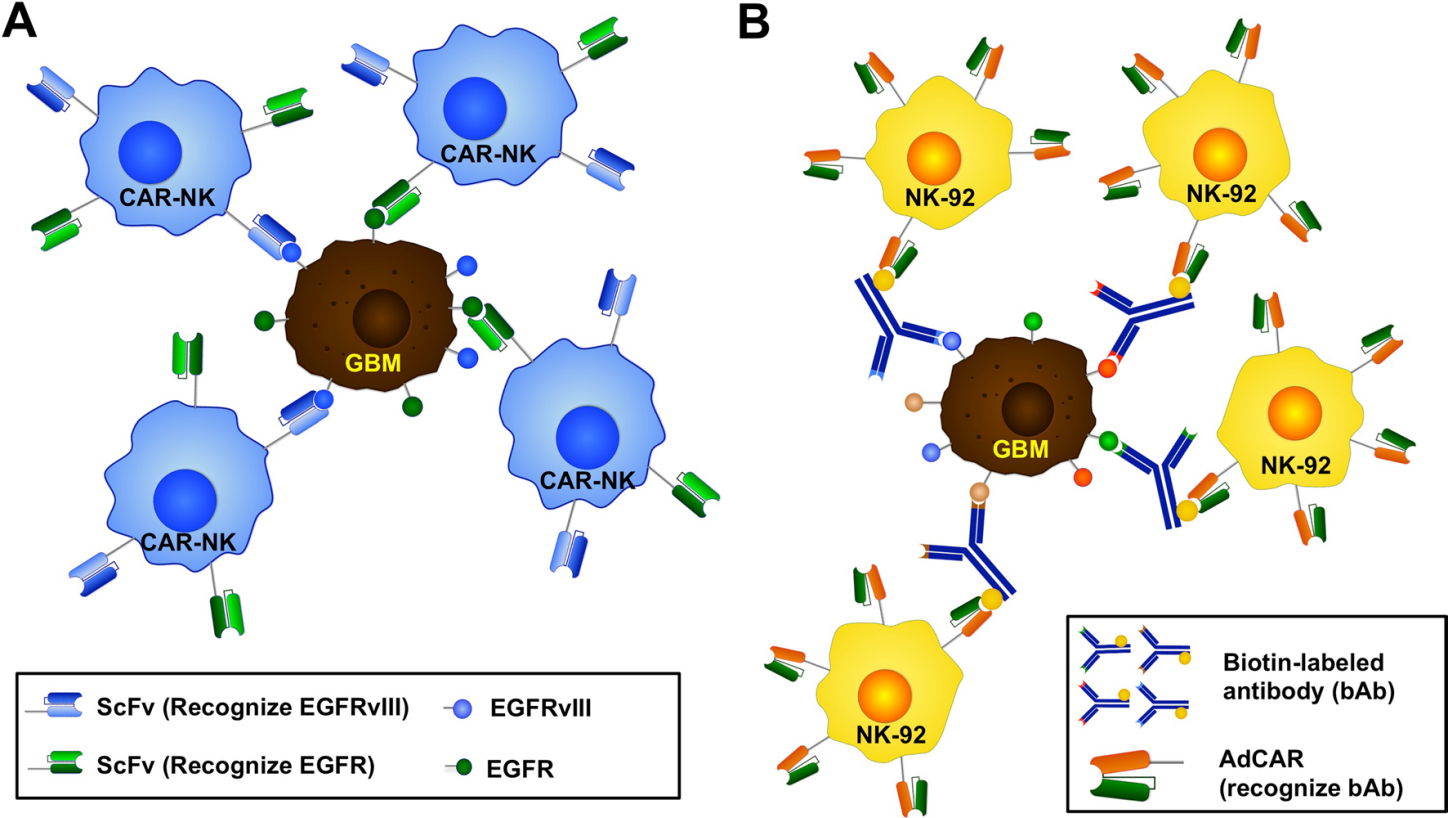
Dual antigen-targeting and adapter chimeric antigen receptor (AdCAR) therapy in glioblastoma (GBM). **A** Structure and recognition of dual antigen-targeting CARs in NK cells. NK cells express scFvs that recognize Epidermal growth factor receptor (EGFR)vIII or EGFR. Therefore, these NK cells can recognize not only GBM cells expressing both EGFRvIII and EGFR but also cells expressing only one of them. **B** AdCAR therapy in GBM. AdCAR can recognize bAb. AdCAR is designed to recognize bAb and is used in conjunction with bAb, which can recognize cancer surface antigens. The important thing is that once AdCAR NK cells are made, it is possible to control them by simply changing bAb, even if the surface antigen of the patient’s cancer changes. Therefore, the use of AdCAR brings flexible and economical benefits for patient-specific immunotherapy. Please see the main text for details

Today, despite significant improvements, more than half of GBM patients treated by single-targeting chimeric antigen receptor NK cells have unsatisfied clinical outcomes. Thereby, to improve the anticancer activity and survivability in patients with GBM, several antigens can be targeted at once, which in preclinical investigations has already yielded encouraging results. Current clinical trials will reveal their potential for the treatment of GBM patients [20, 21]. In other words, dual-antigen targeting can be applied to CAR NK cells against a set of antigens that are present on cancer cells at a higher rate but exhibit limited expression on normal cells to provide enhanced tumor selectivity and NK cell-mediated target cell disruption [21]. For instance, in immunodeficient mice with GBM presenting EGFR, EGFRvIII positive, or both receptors, it is desirable to integrate additional targets within the target range in engineered NK cells [22, 23]. Genetically engineered NK cells expressing CARs with scFv antibody fragments that simultaneously target both antigens EGFR, EGFRvIII, or an epitope common to both antigens, is preferable to treatment with corresponding one-targeting CAR NK cells (Fig. 2 A) [20, 21]. In general, the EGFRvIII mutant is seen in 20–40% of EGFR-amplified malignancies [24, 25].

Genßler et al. demonstrated that, in mouse models, intracranial injection of CAR targeting both EGFR and EGFRvIII led to significant tumor growth inhibition and greatly extended survival [20]. In another study, Wang et al. developed dual-antigen targeting NK cells with an extra functional feature that protects NK cell activity from being inhibited by immunosuppressive factors produced by the GBM tumor microenvironment (TME) [4]. They designed dual-functional CARs that target disialoganglioside (GD2) and natural killer group 2, member D ligands (NKG2DL), which are broadly present in glioma cells [26–28]. Moreover, the simultaneous local release of anti-CD73 fragments capable of binding to CD73 + GBM TME by proteinase activity in the TME resulted in significantly lower adenosine production rates [29, 30]. In contrast, CD73-induced accumulation of adenosine in TME leads to remarkable impairment of purinergic signaling of NK cells, which further affects their normal functions [31, 32]. Despite significant tumor heterogeneity and various immunosuppressive features of the GBM TME, these multifunctional equipped CAR-NK cells have shown an effective anti-GBM activity, in which tumor progression was either delayed or prevented in all treated mice [19]. However, numerous questions such as longer-term efficacy and the impact of antigen targeting on the immunological landscape of GBM tumors must be resolved before employing NK cell-based GBM immunotherapy in clinical contexts [19].

## Adapter chimeric antigen receptor (AdCAR)

Most current CAR therapeutic interventions target a single tumor antigen, which limits CAR-T or CAR-NK cells as a potential therapeutic approach for cancers that are heterogeneous and phenotypically flexible. This may also explain why the CAR technique has limited anticancer efficacy in patients with solid tumors. Recently, from its first to fourth generation, CAR design has had continual modifications that have enhanced CAR NK cell expansion, cytotoxicity, and cytokine production [32]. AdCAR was created to target a single CAR to multiple kinds of cancer antigen, which simplifies the therapy procedure and therapeutic choices. Moreover, AdCARs increase the tumor selectivity, flexibility, and stability of conventional CAR NK cells [33, 34]. To meet these aims, the target antigen recognition and signaling components of traditional CARs were split, generating a dichotomous system composed of an AdCAR and tumor-specific adapter molecules (AMs) [35]. This dichotomous system has several advantages over conventional CAR systems, including (1) the anti-tumor reactions reduce and disappear with the removal of the AM from the patient and (2) repeated AM administration allows for the restarting of treatment against the same or a different target in the event of cancer recurrence [32, 36]. AMs themselves consist of two active domains (an antigen-binding domain and a CAR binding domain), which serve as a linker between the tumor and AdCAR NK cells and can navigate CAR-NK cells to different tumor-associated antigens (TAA) [35, 37]. In this strategy, when the AM binds to a cancer cell, the AdCAR will attach to it and begin destroying the cancer cells. Three primary types of AdCAR platforms are often used in various types of cancer, including Fc-binding AdCARs, tag-specific AdCARs, and bispecific antibody (bsAb)-binding AdCARs [38–40]. In principle, tag-specific AdCARs are coupled with AMs using chemically and enzymatically bound tags. Most interactions with tag-specific AdCARs occur between avidin or streptavidin conjugated to biotin as well as scFv-based fluorescein isothiocyanate (FITC) CARs binding the synthetic dye FITC [38]. Recently, numerous studies have shown promising results in patients with GBM. For instance, Grote et al. demonstrated that AdCAR engineered NK-92 cell, a human natural killer cell line, using biotinylated antibodies (bAb) as the AM could significantly improve glioma cell lysis, specifically in GBM cancer stem (-like) cells (CSCs) (Fig. 2B) [41]. Notably, AdCAR-mediated lysis of CSCs was substantially higher than AdCAR-mediated lysis of adult GBM cells due to up-regulation of several target antigens on GBM CSCs. They designed a targeting biotinylated monoclonal antibody (mAb) with a novel scFv based on avidin and streptavidin as the AM [41]. The scFv could attach up to four biotin ligands at the same time, for which immune cell activity may be controlled quantitatively (on/off switch) as well as qualitatively (change in target antigen composition) [41, 42]. AdCAR-NK cells, in conjunction with biotinylated therapeutic antibodies, can be utilized for flexible, patient-specific immunotherapy, enabling cancer patients to have access to more economical, safe treatments.

## Killer cell immunoglobulin-like receptors (KIRs) as key immunotherapeutic molecules

**Fig. 3:**
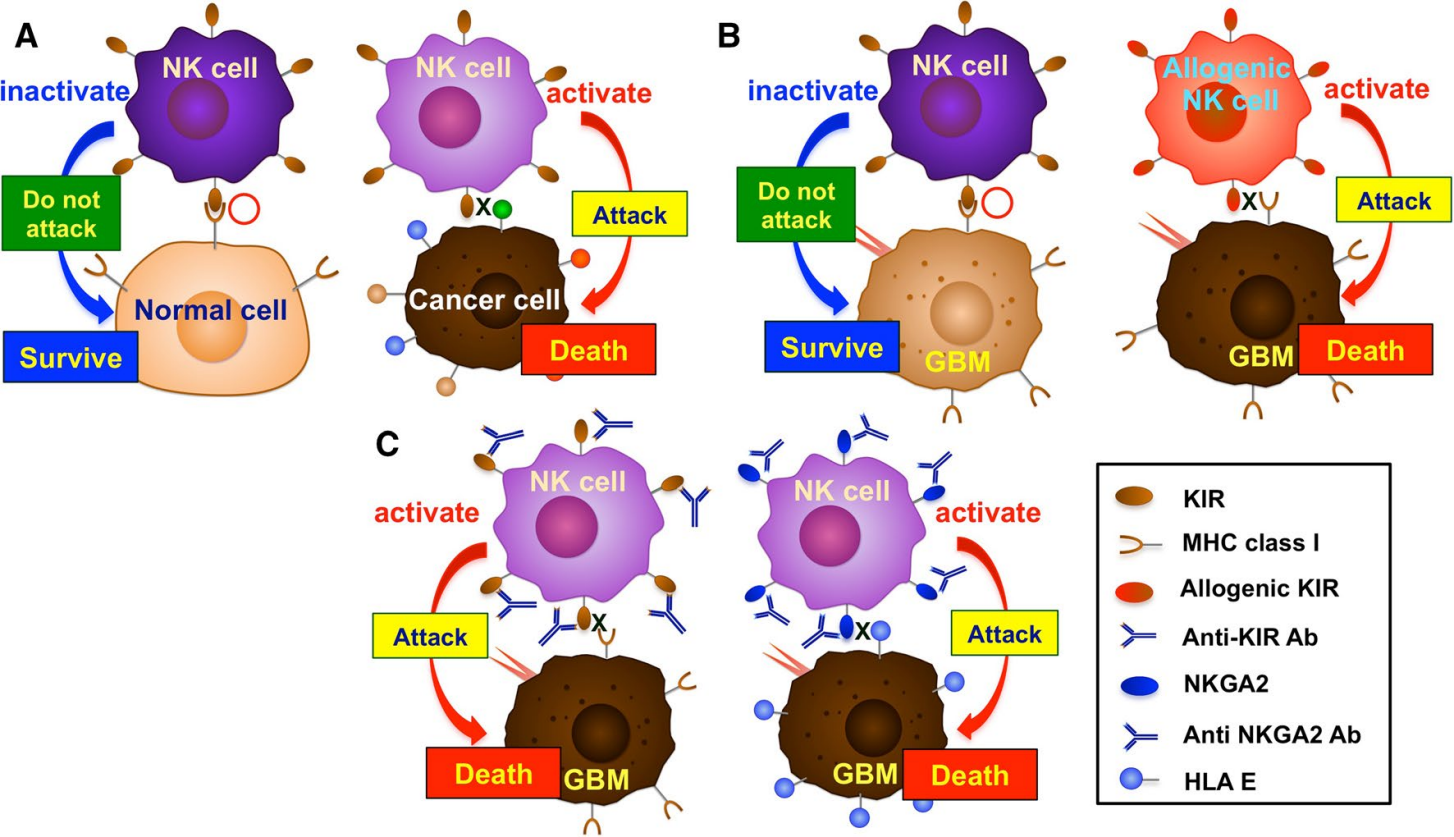
Killer cell immunoglobulin-like receptors (KIR) in NK cells. **A** Normal cells express major histocompatibility complex (MHC) class I molecules such as human leukocyte antigen class I. This binds to KIR, which is expressed on the surface of NK cells. When KIR binds to MHC class I molecules, NK cells recognize cells as autologous, and NK cells are inactivated. Most cancer cells do not express enough HMC class I molecules. Therefore, NK cells can not recognize these cancer cells as autologous cells and attack them. **B** GBM cells express a sufficient amount of MHC class I molecules and are not attacked by NK cells, like normal cells. When allogeneic NK cells were used, they attack GBM because allogeneic NK cells can not recognize GBM cells as autologous cells. **C** Anti-KIR antibody treatment allows KIR to attack GBM because KIR cannot bind to MHC class I molecules. Similarly, Natural killer group 2, member A (NKGA2) antibody treatment leads to activated NK cells. This is because NKGA2 on NK cells cannot bind to HLA-E on GBM, and inhibitory signals do not occur

NK cells are critical components of the innate immune response against invading organisms and cancer cells. NK cells activation, compared to other lymphocytes (T and B cells) is controlled by integrating receptor- and coreceptor-related pathways that involve the interaction of activating and inhibitory receptors on the NK cell membrane with their pair ligands presented on cancer cells [43, 44]. Inhibitory receptors appear in a variety of forms, the most important of which are KIRs, highly conserved NKG2A/CD94 receptors (C-type lectin receptors), and programmed cell death protein 1 (PD-1) [44–46]. Inhibitory signals mediated by KIR are the key regulators of NK cell activation. The primary ligands for these receptors are the human leukocyte antigen class I (HLA-I; HLA-A, B, C) molecules, which are expressed on the surface of normal cells, identifying them as self and, thus, avoiding autoimmunity [44, 46, 47]. In contrast, tumor cells have decreased HLA-I expression, which promotes NK cell activation through activating receptors and subsequent cancer cell killing (Fig. 3A) [44].

Interestingly, rare malignancies such as GBM generate significant quantities of major histocompatibility complex (MHC) class I molecules, which serve as ligands for inhibitory receptors, despite the presence of NK cell-stimulating ligands such as UL-16 binding protein (ULBP) and MICB (ligand for NKG2D), thus evading the NK-mediated regulation of tumor growth and neutralizing the immunoprotective activities of NK cells (Fig. 3B) [48]. Moreover, NK cells make up the smallest percentage of immune cells that infiltrate in glioma (2.11%). Therefore, novel strategies that (1) prevent NK cell inactivation owing to the presence of cancer-related ligands such as HLA-I or (2) increase the amount of NK cells in GBM cancers could significantly improve the anti-tumor activity of NK cells against GBM cancers [48, 49]. Several methods have been developed to enhance NK cell homing in GBM for better treatment (see 5 − 1 and 5 − 2 below).

### Allogeneic NK cells

Adoptive transfer of autologous NK cells is an appealing strategy to expand the number of effective NK cells even in the presence of a high tumor burden. It eliminates concerns about donor-recipient HLA incompatibility and graft rejection by the host immune system [50]. However, autologous NK cell infusion is inefficient in several cancers such as GBM [50], and autologous NK cells are likely to be inhibited by self-HLA signals in tumor cells in the absence of significant activating stimuli or CD16 interaction with antibodies. This circumstance emerges as a consequence of interactions between KIR receptors and their HLA ligands on cancer cells; as a result, NK cell activity is repressed by inhibitory receptors, allowing tumor cell survival [50–53].

To overcome the situation, researchers have continued to examine the use of allogeneic donor NK cells rather than autologous NK cells for cancer treatment [50–53]. Even if the cancer cells (such as GBM) express sufficient amount of MHC class I molecules, allogeneic NK cells can attack GBM because they do not recognize GBM cells as autologous cells (Fig. 3B) [51]. This method has the disadvantage that it is difficult to activate allogeneic NK cells due to immunological incompatibility between tumor MHC molecules and donor KIR expressed on allogeneic NK. However, the presence of several cytokines can activate the allogeneic NK cell. It has been reported that interleukin (IL)-2, 12, IL-15, IL-18, and CCL5 are cytokines that have a role in NK cell activation [54].

There is an association between the use of allogeneic NK cells and graft versus host disease (GVHD), but GVHD can be avoided if the injected product is sufficiently T-cell depleted. Despite this, one study discovered an abnormally high rate of acute GVHD in patients who received allogeneic NK cells. However, researchers believe that GVHD is T cell-mediated rather than NK cell-mediated [55]. One of the difficulties with allogeneic NK cells is that they are sometimes rejected by the recipient’s immune system, limiting their therapeutic use [56, 57]. Prevention of rejection can be accomplished by administering lymphodepleting chemotherapy to the patient before the infusion of NK cells. According to animal research, lymphodepletion chemotherapy may increase the effectiveness of adoptively transplanted NK cells and, hence, the therapeutic outcome [57, 58]. The lymphodepletion conditioning regimen includes two medications that are given preferentially: cyclophosphamide and fludarabine [58].

Several studies have investigated the use of allogeneic NK cells in the treatment of GBM tumors. For instance, Shaim et al. confirmed that utilizing genetically modified allogeneic NK cells with a suppressed αv integrin/transforming growth factor (TGF)-β axis or CRISPR-gene-edited TGF-receptor 2 (TGFBR2) dramatically increased the overall survival in mouse model by effectively targeting both GBM stem cells (GSCs) and non-GSCs [6, 59, 60].

A major source of allogeneic NK cells is umbilical cord blood (UCB), which is commonly used in transplant settings. The potential benefits of UCB-derived NK cells as a ready-to-use source for NK cell immunotherapy over peripheral blood (PB) derived NK cells include (1) higher CD56 bright cell surface density; (2) higher expression of CD11c, CD94/NKG2D, and L-selectin; (3) younger cells that are more capable of proliferating; (4) greater production of interferon (IFN)-γ and tumor necrosis factor (TNF)-α; and (5) higher levels of CD69 when activated with IL-12 and IL-18 [61–63].

IFN-γ production by NK cells as a pluripotent cytokine has important functions in antiviral, anticancer, and immunomodulatory processes and should be regarded as a potentially useful factor for suppressing tumor formation via a various pathway [64, 65]. Additionally, IFN-γ may be involved in the generation of cell cycle inhibitors such as p16 and p21 and in autophagy-mediated apoptosis through the production of reactive oxygen species (ROS) [64, 66, 67]. Contrary to these tumor-suppressing effects of IFN-γ, IFN-γ is also known to increase tumor development, angiogenesis, and the homeostasis response [68]. IFN-γ produced by NK and T cells promotes the production of MHC class I molecules in tumor cells, and it can decrease NK cell function via inhibitory receptor ligation [64, 68]. Not surprisingly, the conflicting biological and pathological consequences of IFN-γ continue to be a major topic of research in the literature.

There are currently numerous clinical trials using UCB NK cells for the treatment of cancer. Recently, several studies have found that TGF as an immune-suppressive cytokine in the tumor microenvironment adversely affected NK cell activity by a inhibition of activating receptors such as NKG2D and NKp46. However, cancer cells’ evasion of NK cells is not limited to reducing activator receptor expression. Higher expression of isoform NKp30c of the activating receptor NKp30 (compared to isoforms NKp30a and NKp30b), which mediates an immunosuppressive signal by generating IL-10, is correlated with a weaker activating signaling and decreased TNF- and IFN-secretion in patients with gastrointestinal stromal tumor [69] and neuroblastoma [70]. Other factors generated by the tumor microenvironment, such as prostaglandin E2, adenosine, L-kynurenine, and picolinic acid can also suppress NK cell activation [71, 72]. To mitigate the adverse effects of TGF-β, Yvon et al. used UCB as a source of allogeneic NK cells with a genetically modified dominant-negative TGF-β receptor II and verified that they could sustain both NKG2D expression and release of IFN-γ in the presence of TGF-β for the identification and lysis of GBM cancer cells [62, 73, 74].

### Therapeutic use of anti-KIR or anti-NKG2A mAbs

IFN-γ generated by NK cells increases MHC class I molecule expression in tumor cells and can reduce NK cell activity via inhibitory receptor ligation, which effectively blocks the stimulation signals of co-engaged activating receptors in the immunological synapse [75]. Thus, one potential strategy for increasing NK cell killing is to disrupt this connection with checkpoint blockers, such as monoclonal antibodies that target KIRs or NKG2A (Fig. 3C) [76, 77]. Unprecedented advances in tumor management have been made possible by therapeutic mAb-mediated masking of inhibitory ‘‘checkpoint” receptors (PD-1, KIRs, and NKG2A) [75, 78]. The inhibition of these checkpoints by monoclonal antibodies alleviates the immune cells from suppression and allows them to identify and kill cancer cells [79]. Monalizumab, a humanized immunoglobulin G (IgG) 4, targets NKG2A receptors and inhibits their interaction with HLA-E-expressing cancer cells and enhances degranulation and IFN-γ production by NKG2A + NK cells. Monalizumab is typically administered in combination with other agents such as anti-programmed cell death ligand 1 (PD-L1) blockade, which promotes both T and NK cell immunity [80, 81]. Lirilumab (IPH2102), a human mAb aimed at preventing the interaction of three inhibitory KIRs (KIR2DL-1, -2, and − 3) and their ligands HLA-C1 and C2, not only increased cell-mediated lysis but also augmented antibody-dependent cellular cytotoxicity (ADCC) in clinical trials [82]. Pembrolizumab, nivolumab, and pidilizumab are humanized monoclonal antibodies that target PD-1 as immunological checkpoints on the surface of NK cells and have made immunotherapy an attractive option in GBM compared to conventional treatments [83–85]. However, compared to bevacizumab (anti-vascular endothelial growth factor monoclonal IgG_1_ antibody), use of these agents as a monotherapy, such as nivolumab monotherapy, did not enhance overall survival in patients with GBM [86]. In Tables 1, we summarize some major therapeutic agents used in monotherapy.

**Table 1:** Major therapeutic agents used in monotherapy

Antibody	Molecular Targets	Cancer	Outcome	References
Monalizumab	NKG2A receptors	GBM, NSLCS, breast cancer, head and neck cancer	Enhanced degranulation and IFN-γ production	van Hall et al. [81]
Lirilumab (IPH2102)	KIRs (KIR2DL-1, -2, and − 3)	Breast cancer, GBM, kidney or ovaries hematologic malignancies	Increased cell-mediated lysis, augmenting ADCC	Benson et al. [82]
Nivolumab + Adjuvant	PD1 receptor	Melanoma, NSCLC, GBM, Hodgkin lymphoma, renal cell cancer, and other cancers	High survival rate, activated local and systemic immune responses	Redman et al. [84]
Pembrolizumab	PD1 receptor	Melanoma, NSCLC, GBM, Hodgkin lymphoma, and renal cell cancer	Durable antitumor activity, long-term treatment	Khoja et al. [83]
Pidilizumab	PD1 receptor,	Haematological malignancies, NSCLC, lymphoma, and other cancers	Increased GBM tumor cell destruction,	Fried et al. [85]
	Delta-like 1 (DLL1)		Increased overall survival	

So far, these agents as monotherapy have not proven to be clinically helpful in patients with GBM [87, 88]. The multiplicity of immunosuppressive pathways identified in GBM may require a combination of various immune checkpoint blockers to gain optimal therapeutic outcomes [89]. A recently published study demonstrated that a combination of lirilumab with pembrolizumab or nivolumab with ipilimumab as “dual immunotherapy” for patients with non-small-cell lung carcinoma or melanoma, respectively, had additive effects promoting both ADCC and the response rate in comparison with monotherapy [47, 90]. Moreover, in tumors expressing both HLA-E and PD-L1, inhibition of the NKG2A and PD-1/PD-L1 axis simultaneously resulted in not only increased NK and T cell cytotoxicity but also promoted T cell proliferation and development of T cell memory [89]. In a preclinical trial in an in vivo mouse model, the anti-KIR lirilumab in conjunction with the immunomodulator lenalidomide showed a moderate effect on multiple myeloma tumors when compared to each drug individually [82]. When novel treatments are introduced, combination therapy may also allow the use of more toxic traditional anti-tumor drugs at lower dosages. Patients with compromised immune systems as a result of prior radiation or chemotherapy may also benefit from NK cell augmenting treatments. Overall, a number of clinical trials are now underway, both as monotherapy and in combination with other agents, and have yielded promising results in GBM patients [89].

## NK cell activation via CD16

**Fig. 4:**
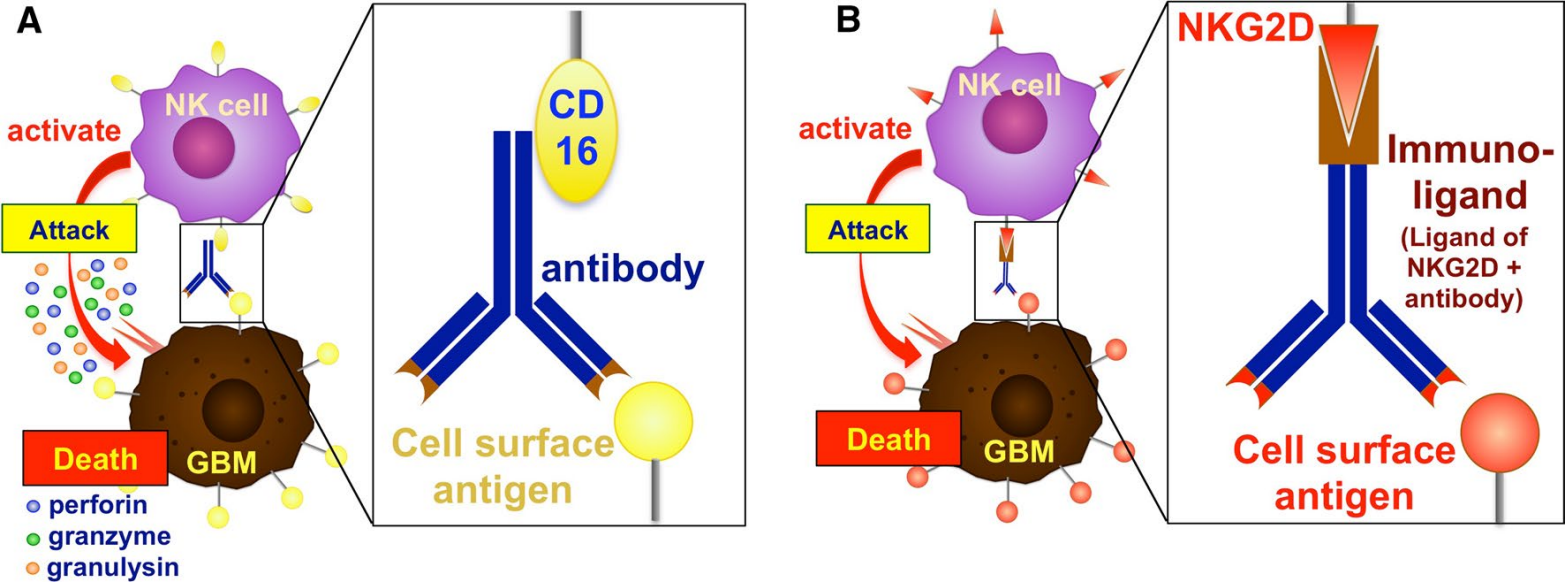
Activation of NK cells via CD16 and immunoligands conjugated to NKG2D. **A** NK cell activation by CD16. CD16 binds to the Fc domain of the antibody that recognizes the cell surface antigens of GBM by its Fab domain. The interaction of CD16 and antibody stimulates the release of cytotoxic granules such as perforin, granulysin and granzyme. **B** NK cell activation by immunoligands. Immunoligands are composed of two domains. One is an antibody (immuno-) domain, which recognizes cell surface antigens of GB cells, and the other is a ligand domain, which can bind to NKG2D on the NK cell surface. When immunoligands recognize GBM via the immunodomain and the ligand domain binds to NKG2D, the NK cells become activated. Activated NK cells attack GBM cells. Currently, immunoligands that simultaneously incorporate two or more antigens have been developed so that NK cells can recognize tumor cells more efficiently and produce cytokines. Please see the main text for details

ADCC is mainly accomplished by immune cells that express surface Fc receptors (such as CD16 or FcγRIIIa) against antigens expressed on target cells that are bound by a particular antibody. ADCC highly depends on IgG antibody subclasses generated by B cells, such as IgG1 and IgG3. Through the employment of an immunologic synapse, this interaction between the Fc domain of these antibodies and CD16 receptors on the surface of immune cells typically stimulates effector cells to release cytotoxic granules carrying perforin, apoptosis-inducing granzymes and granulysin into the target cell, leading to tumor cell lysis (Fig. 4A). Perforin is a cytolytic pore-forming protein that is stored in specialized secretory granules in NK cells and functions by piercing the plasma membrane of the target cells in a Ca^2+^-dependent manner, resulting in cell death. Furthermore, perforin promotes the passive diffusion of serine proteases known as granzymes into the cytosol, which triggers the death signaling pathway through caspase cascade activation [91]. The class of human granzymes consists of 5 members: granzyme A, B, H, K, and M [92]. In Table 2, we summarize the characteristics of each granzyme, separately. Granulysin is an antimicrobial protein that kills intracellular pathogens. Granulysin express in most mammals except for rodents, and the expression is restricted to cytotoxic immune cells such as T cells, NK cells, and NKT cells. Granulysin is active against a broad range of microbes including Gram-positive and -negative bacteria, fungi, and parasites [93].

**Table 2:** Characteristics of granzymes

Granzyme	Cleave site	Substrate	Results	References
Granzyme B	Asp residues	Caspase 3, 7	(1) Impairment of mitochondrial membrane integrity	Prager et al. [92]
		BH3-only protein Bid	(2) Release of apoptotic factors such as cytochrome C	
Granzyme A	Arg or Lys residues	ER-associated SET complex	Causes a new type of cell death known as “athetosis”, which is dependent upon the actin cytoskeleton	Prager et al. [92]
		HMG-2, lamins		
		Histone H1		
Granzyme K	Arg or Lys residues	NAP1, hnRNP K	Production of ROS, chromatin condensation, induction of single-stranded DNA nicks	Prager et al. [92]
Granzyme M	Met or Leu residues	NPM (nucleophosmin), serpin B9 (SP9)	Promotion of granzyme B-induced apoptosis	Prager et al. [92]
Granzyme H	Hydrophobic amino acid residues (Phe or Tyr)	Adenovirus DNA-binding protein, cellular phosphoprotein La	Mitochondrial depolarization, DNA degradation, and chromatin condensation	Prager et al. [92]

Moreover, the stimulation of anti-tumor immune function by immune cells results in the production of cytokines and chemokines such as IFN-γ and granulocyte-macrophage colony-stimulating factor [94]. In this context, NK cells and their receptors have been proven to play a significant role.

NK cells are believed to be the primary mediators of ADCC because they do not co-express the inhibitory FcRγIIb (i.e., the receptor for the Fc region of IgG). Given that CD16 mediates ADCC of NK cells, which specifically recognizes the Fc portion of antibodies, ADCC is considered the primary mechanism of several NK-mediated tumor therapies, such as Pritumumab. Pritumumab is a humanized IgG1 kappa antibody that is designed to block ectodomain vimentin on the surface of glioma cells. It can be identified by NK cells via CD16 receptors and elicits a powerful activating signal that is stronger than the inhibitory signals and stimulates a cytotoxic reaction. Pritumumab has resulted in a safe and effective therapy at doses 5–10 fold less than currently approved antibodies in the treatment of GBM patients in restricted clinical trials [95]. Other studies showed that a mouse mAb called 8B6, which is specific for the O-acetyl GD2 that is abundantly expressed in glioma cells, dramatically limited GBM-bearing mice cell proliferation in vitro and in vivo [96–98]. Particularly, NK-mediated ADCC has been linked to the clinical effectiveness of anti-ganglioside antibodies [96]. Similarly, other studies demonstrated that targeting of neuron glial antigen 2 or chondroitin sulfate proteoglycan 4 (NG2/CSPG4) with the monoclonal antibody mAb9.2.27 could inhibit tumor growth and prolong survival in GBM-bearing mouse and rat models [99, 100]. The expression of NG2/CSPG4 has been observed in vascular pericytes, oligodendroglioma, endothelial cells, and a subpopulation of GBM cells [100]. According to a recent study, 50% of GBM patients’ biopsies exhibit high levels of NG2/CSPG4 expression, which is linked to *EGFR* gene amplification (*p* = 0.0005) and a worse prognosis [101]. Additionally, the humanized anti-EGFR cetuximab in conjunction with immune checkpoint blockers such as anti-NKG2A antibody (monalizumab) can dramatically improve NK cell anti-tumor activity against glioma cells, notably in individuals resistant to temozolomide [80].

A significant issue involved in the ADCC process is the shedding of CD16 receptors by a process mediated by matrix metalloproteinase (encoded by *ADAM17*) following activation by an IgG-opsonized target cell or exposure to IL-2 [102]. The use of metalloproteinase inhibitors is a novel method for preserving the expression of CD16, resulting in not only prolonged CD16 signaling but also increased target cell death and cytokine production [103]. Notably, due to the different affinities of IgG subtypes to CD16A receptors, Fc-based antibody formats are facing multiple challenges that should be considered. The development of bispecific or trispecific antibodies is another immunoglobulin-based strategy for increasing CD16A binding affinity and allowing CD16A binding irrespective of patient genotype. Bispecific antibodies (bsAbs) are antibodies that can attach to two distinct targets or epitopes at the same time [104]. Bispecific antibodies are available in a variety of formats, including fragment-based formats, which are formed by combining two different antigen-binding sites without the Fc portion [105], symmetric formats formed by incorporating two antigen-binding sites in regular antibody molecules while retaining the Fc region [104], and asymmetric formats, which result from strategies that force correct HL chain pairing or promote H chain heterodimerization [106]. In one approach, Pogge von Strandmann et al. designed a bispecific antibody by incorporating NKG2DL (i.e., UL16 binding protein (ULBP) 2) with an scFv targeting CD138, which is overexpressed in a variety of cancers. This bsAb showed high efficiency in inducing ADCC and cytokine production against cancer cells [107]. While progress in this sector has been encouraging, most malignancies continue to be resistant to immunotherapy, and more advances are needed for more effective therapy. One promising approach to optimizing the efficacy of immunotherapy is to redirect the cytotoxic activity of effector T cells to specifically eliminate tumor cells [108]. The trispecific antibody platform has made it feasible to use one antibody to engage not only three distinct epitopes on the surface of tumor cells but also T lymphocyte cell-surface molecules and guide these activated T lymphocytes exclusively to cancer cells, which results in improved lysis and protection against tumor growth. Bispecific antibodies have also shown some efficiency in this area [109, 110].

## Immunoligands conjugated to a natural killer group 2, member D (NKG2DL) receptor on NK cells

The absence of MHC class I on cancer cells results in incomplete activation of NK cells. The full anticancer immunosurveillance activity of NK cells is controlled by a complicated interaction of stimulatory and inhibitory cell surface receptors. The primary activating receptors are NKG2D, DNAX accessory molecule 1 (DNAM-1/CD226), FcγRIIIa (CD16a), NKp44, and NKp46. Two classes of NKG2DL are not present on normal cells but are up-regulated in cancers: MHC class I chain-related protein (MIC) A and B and ULBPs. However, individual activating receptor ligation (with the exception of FcγRIIIa) is typically inadequate to induce cytotoxicity or cytokine release in naive NK cells [111]. Cytokines have essential roles in regulating all aspects of NK cell immunological responses. Cytokines such as IL-2, IL-4, IL-7, IL-9, IL-15, IL-18, IL-21, and IFN-α have been shown to enhance the signaling from activating receptors and are suppressed by immunosuppressive cytokines such as TGF-β [112, 113]. The interplay of cytokines and other stimuli causes NK cells to become fully activated. The significance of cytokine combination therapy in NK cell survival in vivo was originally established by the adoptive transfer of NK cells exposed to IL-12, IL-15, and IL-18, which resulted in enhanced cytotoxicity and survival of NK cells against tumors, whereas IL-2 or IL-15 alone was unsuccessful [114]. Moreover, studies have shown that combining IL-12 with IL-2 or IL-15 increased NK cell activation and IFN-γ and TNF-α production, and TNF-α subsequently triggered NK cell death [112, 115]. This type of NK cell death might be a homeostatic mechanism to suppress NK cell function.

Interestingly, during tumor progression, cancer cells experience immunoediting processes and may remove the surface expression of NKG2DL to evade recognition by NK cells. This immunoediting mechanism can result in ligand proteolytic cleavage or down-modulation of NKG2DL in response to cancer cell-derived TGF. As a result, methods that preserve or restore NK cell detection of tumors may provide a novel therapeutic option. In most invasive tumors, surface expression of NKG2DL can be decreased by exosomal excretion or metalloproteinase activity [116]. In mice, antibodies that inhibit the loss of NKG2DL improve NK cell antitumor efficacy [117]. Soluble human NKG2DL produced by this proteolytic cleavage has also been demonstrated to inhibit NK cell antitumor immunity [118]. Another promising strategy for compensating for the lack of ligands associated with activating receptors such as NKG2D is to create recombinant immunoligands that contain antibody fragments targeting antigens that are commonly overexpressed on cancer cells or the extracellular domain of ligands of the NKG2D receptor to interact with host NK cells (Fig. 4B). In prostate cancer, Jachimowicz et al. used an immunoligand that targets the tumor-associated prostate-specific membrane antigen (PSMA) on the surface of cancer cells and tried to activate host NK cells. They used the immunoligand fused with the NKG2D-specific ligand ULBP2 to activate the receptor NKG2D and obtained high anticancer efficacy in the mouse model by inhibiting tumor development in vitro and by increasing cancer cell lysis [119]. In a similar study in colon cancer, a cross-reactive recombinant immunoligand was fused with human ULBP2 and an antibody-derived single chain that binds to carcinoembryonic antigen on the surface of cancer cells. This specifically provided an anti-tumor activity to NK cells [120–122]. By binding to both tumor cells and NK cells, this immunoligand directed NK cells toward cancer cells, independently of NK ligand expression or MHC-I expression, and triggered NK cell-mediated cellular cytotoxicity against cancer cells. Additionally, immunoligand therapy was shown to decrease tumor development dramatically in a syngeneic colon carcinoma animal model [122]. The efficiency of stimulating receptor signaling is determined by several factors, including the concentration and availability of target antigens as well as morphological flexibility [123]. As a result, it is preferable to design immunoligands as multivalent molecules with higher affinity for cell surface antigens and with lower dissociation rates than monovalent molecules, which can rapidly detach from the target antigen. Additionally, oligomerization of the immunoligand leads to more effective cross-linking of effector cells due to improved affinity, resulting in more efficient NK cytokine production [123]. Multivalent immunoligands are synthetic molecules that incorporate two or more antigens either by chemistry, integrating two hybridoma cell lines, or utilizing recombinant DNA technology [124]. For instance, Singer et al. developed a novel format of recombinant antibody-derived proteins called the single-chain Fv triplebody (sctb), which targets CD19 by two distal scFvs while the central scFv attracts NK cells via CD16 [125]. Such an sctb was more effective than a bispecific single-chain Fv antibody fragment with identical scFv segments when used in cytotoxicity studies against CD19-positive human leukemia and lymphoma cells [126]. This technique provides a unique immunotherapeutic approach for solid tumors and has potential for other clinical applications [107].

Although the activation of NKG2DL is an attractive method to overcome GBM, it has some problems. It has been reported that lactate dehydrogenase isoform 5 (*LDHi5*/*LDH-A*) produced by the GBM causes an increase of NKG2DL in normal immune cells [126]. This prevents these cells from recognizing NKG2DL-bearing tumors, which impairs their ability to attack GBM. To overcome this problem, it has been suggested that down-regulation of *LDHi5* in GBM might be effective because it would improve the recognition of GBM by NK cells and increase the possibility of eliminating GBMs [126]. Furthermore, it has been reported that NK cells are activated by the shed form of mouse UL16-binding protein-like transcript 1 (MULT1), which has a high affinity for NKG2D [127]. This result indicates that a soluble immunoligand also can activate NK cells. Therefore, activation of the soluble ligand such as MULT1 shed form might be one of the keys to future therapeutic strategies for GBM treatment.

## Conclusion

GBM is one of the worst-prognosis tumors, but recent immunotherapies have improved the outcomes of GBM. Further investigation will be needed to overcome GBM, but as with other solid tumors, immunotherapy by NK cells might be one of the powerful tools to treat this refractory cancer.

## Data Availability

All data we used in this work can be found in the references.

## Abbreviations

AdCAR: Adapter chimeric antigen receptor
ADCC: Antibody-dependent cellular cytotoxicity
AMs: Adapter molecules
bsAb: Bispecific antibody
CAR-NK cells: Chimeric antigen receptor-Natural Killer cells
CD28: Cluster of Differentiation 28,
CSC: Cancer stem like cells
DAP12: DNAX-activating protein 12
DNAM-1/CD226: DNAX accessory molecule 1
EGFR: Epidermal growth factor receptor
FASL: FAS ligand.
FITC: Fluorescein isothiocyanate
GBM: Glioblastoma
GD2: Disialoganglioside
GSCs: GBM stem cells
GVHD: Graft versus host disease
HER2/ErbB2: Human epidermal growth factor receptor 2
HLA-I: Human leukocyte antigen class I (such as HLA-A, B, C)
IFN: Interferon
IgG: Immunoglobulin G
IL: Interleukin
KIR: Killer cell immunoglobulin-like receptors
LDHi5/LDH-A: Lactate dehydrogenase isoform 5;
mAb: Monoclonal antibody
MHC: Major histocompatibility complex
MIC: MHC class I chain-related protein
MULT1: Mouse UL16-binding protein-like transcript 1
NG2/CSPG4: Targeting neuron glial antigen 2 (also known as chondroitin sulfate proteoglycan 4)
NK cells: Natural killer cells
NKG2DL: Natural killer group 2, member D ligands
PB: Peripheral blood
PD-1: Programmed cell death protein 1
PD-L1: Programmed cell death ligand 1
ROS: Reactive oxygen species
scFv/FRP5: Single-chain variable fragment
sctb: Single-chain Fv triplebody
TGF: Transforming growth factor
TME: Tumor microenvironment
TNF: Tumor necrosis factor
UCB: Umbilical cord blood
ULBP: UL16 binding protein

## References

[1] Wirsching HG, Galanis E, Weller M (2016). Glioblastoma. Handb Clin Neurol..

[2] Stupp R, Mason WP, van den Bent MJ, Weller M, Fisher B, Taphoorn MJ, Belanger K, Brandes AA, Marosi C, Bogdahn U, Curschmann J, Janzer RC, Ludwin SK, Gorlia T, Allgeier A, Lacombe D, Cairncross JG, Eisenhauer E, Mirimanoff RO (2005). European Organisation for Research and Treatment of Cancer Brain Tumor and Radiotherapy Groups; National Cancer Institute of Canada Clinical Trials Group. Radiotherapy plus concomitant and adjuvant temozolomide for glioblastoma. N Engl J Med.

[3] Stupp R, Taillibert S, Kanner A, Read W, Steinberg D, Lhermitte B, Toms S, Idbaih A, Ahluwalia MS, Fink K, Di Meco F, Lieberman F, Zhu JJ, Stragliotto G, Tran D, Brem S, Hottinger A, Kirson ED, Lavy-Shahaf G, Weinberg U, Kim CY, Paek SH, Nicholas G, Bruna J, Hirte H, Weller M, Palti Y, Hegi ME, Ram Z (2017). Effect of tumor-treating fields plus maintenance temozolomide vs maintenance temozolomide alone on survival in patients With glioblastoma: a randomized clinical trial. JAMA.

[4] Wang X, Lu J, Guo G, Yu J (2021). Immunotherapy for recurrent glioblastoma: practical insights and challenging prospects. Cell Death Dis.

[5] Burger MC, Zhang C, Harter PN, Romanski A, Strassheimer F, Senft C, Tonn T, Steinbach JP, Wels WS (2019). CAR-engineered NK cells for the treatment of glioblastoma: turning innate effectors into precision tools for cancer immunotherapy. Front Immunol.

[6] Daher M, Rezvani K (2018). Next generation natural killer cells for cancer immunotherapy: the promise of genetic engineering. Curr Opin Immunol.

[7] Marofi F, Al-Awad AS, Sulaiman Rahman H, Markov A, Abdelbasset WK, Ivanovna Enina Y, Mahmoodi M, Hassanzadeh A, Yazdanifar M, Stanley Chartrand M, Jarahian M (2021). CAR-NK Cell: A New Paradigm in Tumor Immunotherapy. Front Oncol.

[8] Chiossone L, Dumas PY, Vienne M, Vivier E (2018). Natural killer cells and other innate lymphoid cells in cancer. Nat Rev Immunol.

[9] Miller JS, Lanier LL (2019). Natural killer cells in cancer immunotherapy. Ann Rev Cancer Biol.

[10] Oei VYS, Siernicka M, Graczyk-Jarzynka A, Hoel HJ, Yang W, Palacios D, Almåsbak H, Bajor M, Clement D, Brandt L, Önfelt B, Goodridge J, Winiarska M, Zagozdzon R, Olweus J, Kyte JA, Malmberg KJ (2018). Intrinsic functional potential of NK-cell subsets constrains retargeting driven by chimeric antigen receptors. Cancer Immunol Res.

[11] MacKay M, Afshinnekoo E, Rub J, Hassan C, Khunte M, Baskaran N, Owens B, Liu L, Roboz GJ, Guzman ML, Melnick AM, Wu S, Mason CE (2020). The therapeutic landscape for cells engineered with chimeric antigen receptors. Nat Biotechnol.

[12] Hartmann J, Schüßler-Lenz M, Bondanza A, Buchholz CJ (2017). Clinical development of CAR T cells-challenges and opportunities in translating innovative treatment concepts. EMBO Mol Med.

[13] Müller N, Michen S, Tietze S, Töpfer K, Schulte A, Lamszus K, Schmitz M, Schackert G, Pastan I, Temme A (2015). Engineering NK cells modified with an EGFRvIII-specific chimeric antigen receptor to overexpress CXCR4 improves immunotherapy of CXCL12/SDF-1α-secreting glioblastoma. J Immunother.

[14] Beers R, Chowdhury P, Bigner D, Pastan I (2000). Immunotoxins with increased activity against epidermal growth factor receptor vIII-expressing cells produced by antibody phage display. Clin Cancer Res.

[15] Ahmed N, Salsman VS, Kew Y, Shaffer D, Powell S, Zhang YJ, Grossman RG, Heslop HE, Gottschalk S (2010). HER2-specific T cells target primary glioblastoma stem cells and induce regression of autologous experimental tumors. Clin Cancer Res.

[16] Zhang C, Burger MC, Jennewein L, Genßler S, Schönfeld K, Zeiner P, Hattingen E, Harter PN, Mittelbronn M, Tonn T, Steinbach JP, Wels WS (2016). ErbB2/HER2-Specific NK Cells for Targeted Therapy of glioblastoma. J Natl Cancer Inst.

[17] Schönfeld K, Sahm C, Zhang C, Naundorf S, Brendel C, Odendahl M, Nowakowska P, Bönig H, Köhl U, Kloess S, Köhler S, Holtgreve-Grez H, Jauch A, Schmidt M, Schubert R, Kühlcke K, Seifried E, Klingemann HG, Rieger MA, Tonn T, Grez M, Wels WS (2015). Selective inhibition of tumor growth by clonal NK cells expressing an ErbB2/HER2-specific chimeric antigen receptor. Mol Ther.

[18] Grote S, Mittelstaet J, Baden C, Chan KC, Seitz C, Schlegel P, Kaiser A, Handgretinger R, Schleicher S (2020). Adapter chimeric antigen receptor (aCAR)-engineered NK-92 cells: an off-the-shelf cellular therapeutic for universal tumor targeting. Oncoimmunology..

[19] Wang J, Toregrosa-Allen S, Elzey BD, Utturkar S, Lanman NA, Bernal-Crespo V, Behymer MM, Knipp GT, Yun Y, Veronesi MC, Sinn AL, Pollok KE, Brutkiewicz RR, Nevel KS, Matosevic S (2021). Multispecific targeting of glioblastoma with tumor microenvironment-responsive multifunctional engineered NK cells. Proc Natl Acad Sci U S A..

[20] Genßler S, Burger MC, Zhang C, Oelsner S, Mildenberger I, Wagner M, Steinbach JP, Wels WS (2015). Dual targeting of glioblastoma with chimeric antigen receptor-engineered natural killer cells overcomes heterogeneity of target antigen expression and enhances antitumor activity and survival. Oncoimmunology.

[21] Han J, Chu J, Keung Chan W, Zhang J, Wang Y, Cohen JB, Victor A, Meisen WH, Kim SH, Grandi P, Wang QE, He X, Nakano I, Chiocca EA, Glorioso Iii JC, Kaur B, Caligiuri MA, Yu J (2015). CAR-Engineered NK Cells Targeting Wild-Type EGFR and EGFRvIII Enhance Killing of glioblastoma and Patient-Derived glioblastoma Stem Cells. Sci Rep.

[22] Tonn T, Becker S, Esser R, Schwabe D, Seifried E (2001). Cellular immunotherapy of malignancies using the clonal natural killer cell line NK-92. J Hematother Stem Cell Res.

[23] Wels W, Beerli R, Hellmann P, Schmidt M, Marte BM, Kornilova ES, Hekele A, Mendelsohn J, Groner B, Hynes NE (1995). EGF receptor and p185erbB-2-specific single-chain antibody toxins differ in their cell-killing activity on tumor cells expressing both receptor proteins. Int J Cancer.

[24] Aldape KD, Ballman K, Furth A, Buckner JC, Giannini C, Burger PC, Scheithauer BW, Jenkins RB, James CD (2004). Immunohistochemical detection of EGFRvIII in high malignancy grade astrocytomas and evaluation of prognostic significance. J Neuropathol Exp Neurol.

[25] Biernat W, Huang H, Yokoo H, Kleihues P, Ohgaki H (2004). Predominant expression of mutant EGFR (EGFRvIII) is rare in primary glioblastomas. Brain Pathol.

[26] Longee DC, Wikstrand CJ, Månsson JE, He X, Fuller GN, Bigner SH, Fredman P, Svennerholm L, Bigner DD (1991). Disialoganglioside GD2 in human neuroectodermal tumor cell lines and gliomas. Acta Neuropathol.

[27] Eisele G, Wischhusen J, Mittelbronn M, Meyermann R, Waldhauer I, Steinle A, Weller M, Friese MA (2006). TGF-beta and metalloproteinases differentially suppress NKG2D ligand surface expression on malignant glioma cells. Brain.

[28] Weiss T, Schneider H, Silginer M, Steinle A, Pruschy M, Polić B, Weller M, Roth P (2018). NKG2D-dependent antitumor effects of chemotherapy and radiotherapy against glioblastoma. Clin Cancer Res.

[29] Antonioli L, Blandizzi C, Malavasi F, Ferrari D, Haskó G (2016). Anti-CD73 immunotherapy: A viable way to reprogram the tumor microenvironment. OncoImmunology.

[30] Wang J, Matosevic S (2019). NT5E/CD73 as Correlative Factor of Patient Survival and Natural Killer Cell Infiltration in glioblastoma. J Clin Med.

[31] Wang J, Matosevic S (2018). Adenosinergic signaling as a target for natural killer cell immunotherapy. J Mol Med.

[32] Tokarew N, Ogonek J, Endres S, von Bergwelt-Baildon M, Kobold S (2019). Teaching an old dog new tricks: next-generation CAR T cells. Br J Cancer.

[33] Wu C-Y, Roybal KT, Puchner EM, Onuffer J, Lim WA (2015). Remote control of therapeutic T cells through a small molecule-gated chimeric receptor. Science.

[34] Sakemura R, Terakura S, Watanabe K, Julamanee J, Takagi E, Miyao K, Koyama D, Goto T, Hanajiri R, Nishida T, Murata M, Kiyoi H (2016). A Tet-On Inducible System for Controlling CD19-Chimeric Antigen Receptor Expression upon Drug Administration. Cancer Immunol Res.

[35] Seitz CM, Mittelstaet J, Atar D, Hau J, Reiter S, Illi C, Kieble V, Engert F, Drees B, Bender G, Krahl A-C, Knopf P, Schroeder S, Paulsen N, Rokhvarguer A, Scheuermann S, Rapp E, Mast A-S, Rabsteyn A, Schleicher S, Grote S, Schilbach K, Kneilling M, Pichler B, Lock D, Kotter B, Dapa S, Miltenyi S, Kaiser A, Lang P, Handgretinger R, Schlegel P (2021). Novel adapter CAR-T cell technology for precisely controllable multiplex cancer targeting. Oncoimmunology.

[36] Budde LE, Berger C, Lin Y, Wang J, Lin X, Frayo SE, Brouns SA, Spencer DM, Till BG, Jensen MC, Riddell SR, Press OW (2013). Combining a CD20 chimeric antigen receptor and an inducible caspase 9 suicide switch to improve the efficacy and safety of T cell adoptive immunotherapy for lymphoma. PLoS ONE.

[37] Lu YJ, Chu H, Wheeler LW, Nelson M, Westrick E, Matthaei JF, Cardle II, Johnson A, Gustafson J, Parker N, Vetzel M, Xu LC, Wang EZ, Jensen MC, Klein PJ, Low PS, Leamon CP (2019). Preclinical evaluation of bispecific adaptor molecule controlled folate receptor CAR-T cell therapy with special focus on pediatric malignancies. Front Oncol.

[38] Tamada K, Geng D, Sakoda Y, Bansal N, Srivastava R, Li Z, Davila E (2012). Redirecting gene-modified T cells toward various cancer types using tagged antibodies. Clin Cancer Res.

[39] Clémenceau B, Congy-Jolivet N, Gallot G, Vivien R, Gaschet J, Thibault G, Vié H (2006). Antibody-dependent cellular cytotoxicity (ADCC) is mediated by genetically modified antigen-specific human T lymphocytes. Blood.

[40] Stamova S, Koristka S, Keil J, Arndt C, Feldmann A, Michalk I, Bartsch H, Bippes CC, Schmitz M, Cartellieri M, Bachmann M (2012). Cancer immunotherapy by retargeting of immune effector cells via recombinant bispecific antibody constructs. Antibodies.

[41] Grote S, Chan C-H, Baden C, Huber SM, Eckert F, Mittelstaet J, Kaiser A, Christian Seitz C, Patrick Schlegel P, Handgretinger R, Schleicher S (2020). Universal adapter CAR-engineered NK-92 cells target patient-derived glioblastoma cancer stem cells. Cancer Immunol Res.

[42] Urbanska K, Lanitis E, Poussin M, Lynn RC, Gavin BP, Kelderman S, Yu J, Scholler N, Powell DJ (2012). A universal strategy for adoptive immunotherapy of cancer through use of a novel T-cell antigen receptor. Cancer Res.

[43] Campbell KS, Purdy AK (2011). Structure/function of human killer cell immunoglobulin-like receptors: lessons from polymorphisms, evolution, crystal structures and mutations. Immunology.

[44] Hu W, Wang G, Huang D, Sui M, Xu Y (2019). Cancer immunotherapy based on natural killer cells: current progress and new opportunities. Front Immunol.

[45] Béziat V, Liu LL, Malmberg JA, Ivarsson MA, Sohlberg E, Björklund AT, Retière C, Sverremark-Ekström E, Traherne J, Ljungman P, Schaffer M, Price DA, Trowsdale J, Michaëlsson J, Ljunggren HG, Malmberg KJ (2013). NK cell responses to cytomegalovirus infection lead to stable imprints in the human KIR repertoire and involve activating KIRs. Blood.

[46] Pesce S, Greppi M, Tabellini G, Rampinelli F, Parolini S, Olive D, Moretta L, Moretta A, Marcenaro E (2017). Identification of a subset of human natural killer cells expressing high levels of programmed death 1: A phenotypic and functional characterization. J Allergy Clin Immunol.

[47] He Y, Liu S, Mattei J, Bunn PA, Zhou C, Chan D (2018). The combination of anti-KIR monoclonal antibodies with anti-PD-1/PD-L1 monoclonal antibodies could be a critical breakthrough in overcoming tumor immune escape in NSCLC. Drug Des Devel Ther.

[48] Golán I, de la RodríguezFuente L, Costoya JA (2018). NK cell-based glioblastoma immunotherapy. Cancers (Basel).

[49] Kmiecik J, Poli A, Brons NH, Waha A, Eide GE, Enger P, Zimmer J, Chekenya M (2013). Elevated CD3 + and CD8 + tumor-infiltrating immune cells correlate with prolonged survival in glioblastoma patients despite integrated immunosuppressive mechanisms in the tumor microenvironment and at the systemic level. J Neuroimmunol.

[50] Lim O, Jung MY, Hwang YK, Shin E-C (2015). Present and future of allogeneic natural killer cell therapy. Front Immunol.

[51] Leung W (2014). Infusions of allogeneic natural killer cells as cancer therapy. Clin Cancer Res.

[52] Vago L, Forno B, Sormani MP, Crocchiolo R, Zino E, Di Terlizzi S, Lupo Stanghellini MT, Mazzi B, Perna SK, Bondanza A, Middleton D, Palini A, Bernardi M, Bacchetta R, Peccatori J, Rossini S, Roncarolo MG, Bordignon C, Bonini C, Ciceri F, Fleischhauer K (2008). Temporal, quantitative, and functional characteristics of single-KIR–positive alloreactive natural killer cell recovery account for impaired graft-versus-leukemia activity after haploidentical hematopoietic stem cell transplantation. Blood.

[53] Ruggeri L, Capanni M, Urbani E, Perruccio K, Shlomchik WD, Tosti A, Posati S, Rogaia D, Frassoni F, Aversa F, Martelli MF, Velardi A (2002). Effectiveness of donor natural killer cell alloreactivity in mismatched hematopoietic transplants. Science.

[54] Topham NJ, Hewitt EW (2009). Natural killer cell cytotoxicity: how do they pull the trigger?. Immunology.

[55] Shah NN, Baird K, Delbrook CP, Fleisher TA, Kohler ME, Rampertaap S, Lemberg K, Hurley CK, Kleiner DE, Merchant MS, Pittaluga S, Sabatino M, Stroncek DF, Wayne AS, Zhang H, Fry TJ, Mackall CL (2015). Acute GVHD in patients receiving IL-15/4-1BBL activated NK cells following T-cell-depleted stem cell transplantation. Blood.

[56] Miller JS, Soignier Y, Panoskaltsis-Mortari A, McNearney SA, Yun GH, Fautsch SK, McKenna D, Le C, Defor TE, Burns LJ, Orchard PJ, Blazar BR, Wagner JE, Slungaard A, Weisdorf DJ, Okazaki IJ, McGlave PB (2005). Successful adoptive transfer and in vivo expansion of human haploidentical NK cells in patients with cancer. Blood.

[57] Rubnitz JE, Inaba H, Ribeiro RC, Pounds S, Rooney B, Bell T, Pui CH, Leung W (2010). NKAML: a pilot study to determine the safety and feasibility of haploidentical natural killer cell transplantation in childhood acute myeloid leukemia. J Clin Oncol.

[58] Muranski P, Boni A, Wrzesinski C, Citrin DE, Rosenberg SA, Childs R, Restifo NP (2006). Increased intensity lymphodepletion and adoptive immunotherapy–how far can we go?. Nat Clin Pract Oncol.

[59] Castriconi R, Daga A, Dondero A, Zona G, Poliani PL, Melotti A, Griffero F, Marubbi D, Spaziante R, Bellora F, Moretta L, Moretta A, Corte G, Bottino C (2009). NK Cells Recognize and Kill Human glioblastoma Cells with Stem Cell-Like Properties. J Immunol.

[60] Shaim H, Shanley M, Basar R, Daher M, Gumin J, Zamler DB, Uprety N, Wang F, Huang Y, Gabrusiewicz K, Miao Q, Dou J, Alsuliman A, Kerbauy LN, Acharya S, Mohanty V, Mendt M, Li S, Lu J, Wei J, Fowlkes NW, Gokdemir E, Ensley EL, Kaplan M, Kassab C, Li L, Ozcan G, Banerjee PP, Shen Y, Gilbert AL, Jones CM, Bdiwi M, Nunez-Cortes AK, Liu E, Yu J, Imahashi N, Muniz-Feliciano L, Li Y, Hu J, Draetta G, Marin D, Yu D, Mielke S, Eyrich M, Champlin RE, Chen K, Lang FF, Shpall EJ, Heimberger AB, Rezvani K (2021). Targeting the αv integrin/TGF-β axis improves natural killer cell function against glioblastoma stem cells. J Clin Investig.

[61] Veluchamy JP, Kok N, van der Vliet HJ, Verheul HMW, de Gruijl TD, Spanholtz J (2017). The rise of allogeneic natural killer cells as a platform for cancer immunotherapy: recent innovations and future developments. Front Immunol.

[62] Yvon ES, Burga R, Powell A, Cruz CR, Fernandes R, Barese C, Nguyen T, Abdel-Baki MS, Bollard CM (2017). Cord blood natural killer cells expressing a dominant negative TGF-β receptor: Implications for adoptive immunotherapy for glioblastoma. Cytotherapy.

[63] Zhao X, Cai L, Hu Y, Wang H (2020). Cord-blood natural killer cell-based immunotherapy for cancer. Front Immunol.

[64] Mojic M, Takeda K, Hayakawa Y (2017). The dark side of IFN-γ: its role in promoting cancer immunoevasion. Int J Mol Sci.

[65] Ni L, Lu J (2018). Interferon gamma in cancer immunotherapy. Cancer Med.

[66] Chin YE, Kitagawa M, Su WC, You ZH, Iwamoto Y, Fu XY (1996). Cell growth arrest and induction of cyclin-dependent kinase inhibitor p21 WAF1/CIP1 mediated by STAT1. Science.

[67] Chawla-Sarkar M, Lindner DJ, Liu YF, Williams BR, Sen GC, Silverman RH, Borden EC (2003). Apoptosis and interferons: role of interferon-stimulated genes as mediators of apoptosis. Apoptosis.

[68] Jorgovanovic D, Song M, Wang L, Zhang Y (2020). Roles of IFN-γ in tumor progression and regression: a review. Biomark Res.

[69] Delahaye NF, Rusakiewicz S, Martins I, Ménard C, Roux S, Lyonnet L, Paul P, Sarabi M, Chaput N, Semeraro M, Minard-Colin V, Poirier-Colame V, Chaba K, Flament C, Baud V, Authier H, Kerdine-Römer S, Pallardy M, Cremer I, Peaudecerf L, Rocha B, Valteau-Couanet D, Gutierrez JC, Nunès JA, Commo F, Bonvalot S, Ibrahim N, Terrier P, Opolon P, Bottino C, Moretta A, Tavernier J, Rihet P, Coindre JM, Blay JY, Isambert N, Emile JF, Vivier E, Lecesne A, Kroemer G, Zitvogel L (2011). Alternatively spliced NKp30 isoforms affect the prognosis of gastrointestinal stromal tumors. Nat Med.

[70] Semeraro M, Rusakiewicz S, Zitvogel L, Kroemer G (2015). Natural killer cell mediated immunosurveillance of pediatric neuroblastoma. Oncoimmunology.

[71] Frumento G, Rotondo R, Tonetti M, Damonte G, Benatti U, Ferrara GB (2002). Tryptophan-derived catabolites are responsible for inhibition of T and natural killer cell proliferation induced by indoleamine 2,3-dioxygenase. J Exp Med.

[72] Young A, Ngiow SF, Gao Y, Patch AM, Barkauskas DS, Messaoudene M, Lin G, Coudert JD, Stannard KA, Zitvogel L, Degli-Esposti MA, Vivier E, Waddell N, Linden J, Huntington ND, Souza-Fonseca-Guimaraes F, Smyth MJ (2018). A2AR adenosine signaling suppresses natural killer cell maturation in the tumor microenvironment. Cancer Res.

[73] Yang L, Pang Y, Moses HL (2010). TGF-β and immune cells: an important regulatory axis in the tumor microenvironment and progression. Trends Immunol.

[74] Lee Hm, Kim K-S, Kim J (2014). A comparative study of the effects of inhibitory cytokines on human natural killer cells and the mechanistic features of transforming growth factor-beta. Cell Immunol.

[75] Pende D, Falco M, Vitale M, Cantoni C, Vitale C, Munari E, Bertaina A, Moretta F, Del Zotto G, Pietra G, Mingari MC, Locatelli F, Moretta L (2019). Killer Ig-like receptors (KIRs): their role in NK cell modulation and developments leading to their clinical exploitation. Front Immunol.

[76] Braud VM, Allan DS, O’Callaghan CA, Söderström K, D’Andrea A, Ogg GS, Lazetic S, Young NT, Bell JI, Phillips JH, Lanier LL, McMichael AJ (1998). HLA-E binds to natural killer cell receptors CD94/NKG2A, B and C. Nature.

[77] Lazetic S, Chang C, Houchins JP, Lanier LL, Phillips JH (1996). Human natural killer cell receptors involved in MHC class I recognition are disulfide-linked heterodimers of CD94 and NKG2 subunits. J Immunol.

[78] Benson DM, Bakan CE, Zhang S, Collins SM, Liang J, Srivastava S, Hofmeister CC, Efebera Y, Andre P, Romagne F, Bléry M, Bonnafous C, Zhang J, Clever D, Caligiuri MA, Farag SS (2011). IPH2101, a novel anti-inhibitory KIR antibody, and lenalidomide combine to enhance the natural killer cell versus multiple myeloma effect. Blood.

[79] Khan M, Arooj S, Wang H (2020). NK cell-based immune checkpoint inhibition. Front Immunol.

[80] André P, Denis C, Soulas C, Bourbon-Caillet C, Lopez J, Arnoux T, Bléry M, Bonnafous C, Gauthier L, Morel A, Rossi B, Remark R, Breso V, Bonnet E, Habif G, Guia S, Lalanne AI, Hoffmann C, Lantz O, Fayette J, Boyer-Chammard A, Zerbib R, Dodion P, Ghadially H, Jure-Kunkel M, Morel Y, Herbst R, Narni-Mancinelli E, Cohen RB, Vivier E (2018). Anti-NKG2A mAb is a checkpoint inhibitor that promotes anti-tumor immunity by unleashing both T and NK cells. Cell.

[81] van Hall T, André P, Horowitz A, Ruan DF, Borst L, Zerbib R, Narni-Mancinelli E, van der Burg SH, Vivier E (2019). Monalizumab: inhibiting the novel immune checkpoint NKG2A. J Immunother Cancer.

[82] Benson DM, Hofmeister CC, Padmanabhan S, Suvannasankha A, Jagannath S, Abonour R, Bakan C, Andre P, Efebera Y, Tiollier J, Caligiuri MA, Farag SS (2012). A phase 1 trial of the anti-KIR antibody IPH2101 in patients with relapsed/refractory multiple myeloma. Blood.

[83] Khoja L, Butler MO, Kang SP, Ebbinghaus S, Joshua AM, Pembrolizumab (2015). J Immunother Cancer.

[84] Redman JM, Hill EM, AlDeghaither D, Weiner LM (2015). Mechanisms of action of therapeutic antibodies for cancer. Mol Immunol.

[85] Fried I, Lossos A, Ben Ami T, Dvir R, Toledano H, Ben Arush MW, Postovski S, Abu Kuidar A, Yalon M, Weintraub M, Benifla M (2018). Preliminary results of immune modulating antibody MDV9300 (pidilizumab) treatment in children with diffuse intrinsic pontine glioma. J Neurooncol.

[86] Reardon DA, Omuro A, Brandes AA, Rieger J, Wick A, Sepulveda J, de PhuphanichSSouza P, Ahluwalia MS, Lim M, Vlahovic G, Sampson J (2017). Randomized phase 3 study evaluating the efficacy and safety of nivolumab vs bevacizumab in patients With recurrent glioblastoma: checkMate 143. Neurooncology.

[87] Majc B, Novak M, Kopitar-Jerala N, Jewett A, Breznik B (2021). Immunotherapy of glioblastoma: current strategies and challenges in tumor model development. Cells.

[88] Medikonda R, Dunn G, Rahman M, Fecci P, Lim M (2021). A review of glioblastoma immunotherapy. J Neurooncol.

[89] Preusser M, Lim M, Hafler DA, Reardon DA, Sampson JH (2015). Prospects of immune checkpoint modulators in the treatment of glioblastoma. Nat Rev Neurol.

[90] Larkin J, Chiarion-Sileni V, Gonzalez R, Grob JJ, Rutkowski P, Lao CD, Cowey CL, Schadendorf D, Wagstaff J, Dummer R, Ferrucci PF, Smylie M, Hogg D, Hill A, Márquez-Rodas I, Haanen J, Guidoboni M, Maio M, Schöffski P, Carlino MS, Lebbé C, McArthur G, Ascierto PA, Daniels GA, Long GV, Bastholt L, Rizzo JI, Balogh A, Moshyk A, Hodi FS, Wolchok JD (2019). Five-year survival with combined nivolumab and ipilimumab in advanced melanoma. N Engl J Med.

[91] Uellner R, Zvelebil MJ, Hopkins J, Jones J, MacDougall LK, Morgan BP (1997). Perforin is activated by a proteolytic cleavage during biosynthesis which reveals a phospholipid-binding C2 domain. EMBO J.

[92] Prager I, Watzl C (2019). Mechanisms of natural killer cell-mediated cellular cytotoxicity. J Leukoc Biol.

[93] Liu X, Lieberman J (2020). Knocking ‘em dead: pore-forming proteins in immune defense. Annu Rev Immunol.

[94] Qiu JT, Alson D, Lee TH, Tsai CC, Yu TW, Chen YS, Cheng YF, Lin CC, Schuyler SC (2019). Effect of multiple vaccinations with tumor cell-based vaccine with codon-modified GM-CSF on tumor growth in a mouse model. Cancers.

[95] Kesari S, Babic I, Mukthavaram R, Jiang P, Nomura N, Pingle SC, Juarez T, Yang J, Yenugonda V, Nurmemmedov E, Glassy MC (2017). Pritumumab binding to glioma cells induces ADCC and inhibits tumor growth. J Clin Oncol.

[96] Imai M, Landen C, Ohta R, Cheung NK, Tomlinson S (2005). Complement-mediated mechanisms in anti-GD2 monoclonal antibody therapy of murine metastatic cancer. Cancer Res.

[97] Zeng Y, Fest S, Kunert R, Katinger H, Pistoia V, Michon J, Lewis G, Ladenstein R, Lode HN (2005). Anti-neuroblastoma effect of ch14.18 antibody produced in CHO cells is mediated by NK-cells in mice. Mol Immunol.

[98] Fleurence J, Cochonneau D, Fougeray S, Oliver L, Geraldo F, Terme M, Dorvillius M, Loussouarn D, Vallette F, Paris F, Birklé S (2016). Targeting and killing GBM with monoclonal antibody to O-acetyl GD2 ganglioside. Oncotarget.

[99] Poli A, Wang J, Domingues O, Planagumà J, Yan T, Rygh CB, Skaftnesmo KO, Thorsen F, McCormack E, Hentges F, Pedersen PH, Zimmer J, Enger P, Chekenya M (2013). Targeting GBM with NK cells and mAb against NG2/CSPG4 prolongs animal survival. Oncotarget.

[100] Kmiecik J, Gras Navarro A, Poli A, Planagumà JP, Zimmer J, Chekenya M (2014). Combining NK cells and mAb9.2.27 to combat NG2-dependent and anti-inflammatory signals in GBM. OncoImmunology.

[101] Svendsen A, Verhoeff JJ, Immervoll H, Brøgger JC, Kmiecik J, Poli A, Netland IA, Prestegarden L, Planagumà J, Torsvik A, Kjersem AB, Sakariassen P, Heggdal JI, Van Furth WR, Bjerkvig R, Lund-Johansen M, Enger P, Felsberg J, Brons NH, Tronstad KJ, Waha A, Chekenya M (2011). Expression of the progenitor marker NG2/CSPG4 predicts poor survival and resistance to ionising radiation in GBM. Acta Neuropathol.

[102] Peruzzi G, Femnou L, Gil-Krzewska A, Borrego F, Weck J, Krzewski K, Coligan JE (2013). Membrane-type 6 matrix metalloproteinase regulates the activation-induced downmodulation of CD16 in human primary NK cells. J Immunol.

[103] Zhou Q, Gil-Krzewska A, Peruzzi G, Borrego F (2013). Matrix metalloproteinases inhibition promotes the polyfunctionality of human natural killer cells in therapeutic antibody-based anti-tumour immunotherapy. Clin Exp Immunol.

[104] Chames P, Baty D (2009). Bispecific antibodies for cancer therapy: the light at the end of the tunnel?. MAbs..

[105] Birch JR, Racher AJ (2006). Antibody production. Adv Drug Deliv Rev.

[106] Margni RA (1994). Coprecipitating IgG asymmetric antibodies: a possible role for Fab glycosylation, and speculations on their formation and functions in disease. Glycosylation & Disease.

[107] von Strandmann EP, Hansen HP, Reiners KS, Schnell R, Borchmann P, Merkert S, Simhadri VR, Draube A, Reiser M, Purr I, Hallek M, Engert A (2006). A novel bispecific protein (ULBP2-BB4) targeting the NKG2D receptor on natural killer (NK) cells and CD138 activates NK cells and has potent antitumor activity against human multiple myeloma in vitro and in vivo. Blood.

[108] Baeuerle PA, Reinhardt C (2009). Bispecific T-cell engaging antibodies for cancer therapy. Cancer Res.

[109] Sun LL, Ellerman D, Mathieu M, Hristopoulos M, Chen X, Li Y, Yan X, Clark R, Reyes A, Stefanich E, Mai E, Young J, Johnson C, Huseni M, Wang X, Chen Y, Wang P, Wang H, Dybdal N, Chu YW, Chiorazzi N, Scheer JM, Junttila T, Totpal K, Dennis MS, Ebens AJ (2015). Anti-CD20/CD3 T cell-dependent bispecific antibody for the treatment of B cell malignancies. Sci Transl Med.

[110] Zugmaier G, Gökbuget N, Klinger M, Viardot A, Stelljes M, Neumann S, Horst HA, Marks R, Faul C, Diedrich H, Reichle A, Brüggemann M, Holland C, Schmidt M, Einsele H, Bargou RC, Topp MS (2015). Long-term survival and T-cell kinetics in relapsed/refractory ALL patients who achieved MRD response after blinatumomab treatment. Blood.

[111] Bryceson YT, March ME, Ljunggren HG, Long EO (2006). Synergy among receptors on resting NK cells for the activation of natural cytotoxicity and cytokine secretion. Blood.

[112] Zwirner NW, Domaica CI (2010). Cytokine regulation of natural killer cell effector functions. BioFactors.

[113] Abel AM, Yang C, Thakar MS, Malarkannan S (2018). Natural killer cells: development, maturation, and clinical utilization. Front Immunol.

[114] Ni J, Miller M, Stojanovic A, Garbi N, Cerwenka A (2012). Sustained effector function of IL-12/15/18-preactivated NK cells against established tumors. J Exp Med.

[115] Ross ME, Caligiuri MA (1997). Cytokine-induced apoptosis of human natural killer cells identifies a novel mechanism to regulate the innate immune response. Blood.

[116] Baragaño Raneros A, Suarez-Álvarez B, López-Larrea C (2014). Secretory pathways generating immunosuppressive NKG2D ligands: new targets for therapeutic intervention. Oncoimmunology.

[117] Ferrari de Andrade L, Tay RE, Pan D, Luoma AM, Ito Y, Badrinath S, Tsoucas D, Franz B, May KF, Harvey CJ, Kobold S, Pyrdol JW, Yoon C, Yuan GC, Hodi FS, Dranoff G, Wucherpfennig KW (2018). Antibody-mediated inhibition of MICA and MICB shedding promotes NK cell-driven tumor immunity. Science.

[118] Xiao G, Wang X, Sheng J, Lu S, Yu X, Wu JD (2015). Soluble NKG2D ligand promotes MDSC expansion and skews macrophage to the alternatively activated phenotype. J Hematol Oncol.

[119] Jachimowicz RD, Fracasso G, Yazaki PJ, Power BE, Borchmann P, Engert A, Hansen HP, Reiners KS, Marie M, von Strandmann EP, Rothe A (2011). Induction of in vitro and in vivo NK cell cytotoxicity using high-avidity immunoligands targeting prostate-specific membrane antigen in prostate carcinoma. Mol Cancer Ther.

[120] Cao W, He W (2004). UL16 binding proteins. Immunobiology.

[121] Rodenburg CM, Mernaugh R, Bilbao G, Khazaeli MB (1998). Production of a single chain anti-CEA antibody from the hybridoma cell line T84.66 using a modified colony-lift selection procedure to detect antigen-positive ScFv bacterial clones. Hybridoma.

[122] Rothe A, Jachimowicz RD, Borchmann S, Madlener M, Kessler J, Reiners K, Sauer M, Hansen M, Ullrich R, Chatterjee S, Borchmann P, Yazaki P, Koslowsky T, Engert A, Heukamp L, Hallek M, Strandmann EP (2014). The bispecific immunoligand ULBP2-aCEA redirects natural killer cells to tumor cells and reveals potent anti-tumor activity against colon carcinoma. Int J Cancer.

[123] Holliger P, Hudson PJ (2005). Engineered antibody fragments and the rise of single domains. Nat Biotechnol.

[124] Byrne H, Conroy PJ, Whisstock JC, O’Kennedy RJ (2013). A tale of two specificities: bispecific antibodies for therapeutic and diagnostic applications. Trends Biotechnol.

[125] Singer H, Kellner C, Lanig H, Aigner M, Stockmeyer B, Oduncu F, Schwemmlein M, Stein C, Mentz K, Mackensen A, Fey GH (2010). Effective elimination of acute myeloid leukemic cells by recombinant bispecific antibody derivatives directed against CD33 and CD16. J Immunother.

[126] Crane CA, Austgen K, Haberthur K, Hofmann C, Moyes KW, Avanesyan L, Fong L, Campbell MJ, Cooper S, Oakes SA, Parsa AT, Lanier LL (2014). Immune evasion mediated by tumor-derived lactate dehydrogenase induction of NKG2D ligands on myeloid cells in GBM patients. Proc Natl Acad Sci U S A.

[127] Deng W, Gowen BG, Zhang L, Wang L, Lau S, Iannello A, Xu J, Rovis TL, Xiong N, Raulet DH (2015). Antitumor immunity. A shed NKG2D ligand that promotes natural killer cell activation and tumor rejection. Science.

